# A unifying model to estimate thermal tolerance limits in ectotherms across static, dynamic and fluctuating exposures to thermal stress

**DOI:** 10.1038/s41598-021-92004-6

**Published:** 2021-06-18

**Authors:** Lisa Bjerregaard Jørgensen, Hans Malte, Michael Ørsted, Nikolaj Andreasen Klahn, Johannes Overgaard

**Affiliations:** Zoophysiology, Department of Biology, Aarhus University, 8000 Aarhus C, Denmark

**Keywords:** Animal physiology, Ecophysiology, Biogeography, Ecological modelling, Climate-change ecology

## Abstract

Temperature tolerance is critical for defining the fundamental niche of ectotherms and researchers classically use either static (exposure to a constant temperature) or dynamic (ramping temperature) assays to assess tolerance. The use of different methods complicates comparison between studies and here we present a mathematical model (and *R*-scripts) to reconcile thermal tolerance measures obtained from static and dynamic assays. Our model uses input data from several static or dynamic experiments and is based on the well-supported assumption that thermal injury accumulation rate increases exponentially with temperature (known as a thermal death time curve). The model also assumes thermal stress at different temperatures to be additive and using experiments with *Drosophila melanogaster*, we validate these central assumptions by demonstrating that heat injury attained at different heat stress intensities and durations is additive. In a separate experiment we demonstrate that our model can accurately describe injury accumulation during fluctuating temperature stress and further we validate the model by successfully converting literature data of ectotherm heat tolerance (both static and dynamic assays) to a single, comparable metric (the temperature tolerated for 1 h). The model presented here has many promising applications for the analysis of ectotherm thermal tolerance and we also discuss potential pitfalls that should be considered and avoided using this model.

## Introduction

Tolerance to temperature extremes is arguably among the most important traits defining the fundamental niche of ectotherms^1–5^. However, studies of thermal tolerance are difficult to compare due to the plethora of static (constant temperature) and dynamic (ramping temperature) assays used to assess tolerance limits^3,6–19^. Furthermore, it is difficult to relate laboratory measures of thermal tolerance to the temperature stress experienced under natural conditions particularly as the duration and intensity of temperature stress may fluctuate unpredictably.

Static and dynamic methods differ in their temperature protocol (constant and changing temperature, respectively) but also in duration of the stress during testing (see discussions in^9,13,15,20^). A solution to unify many of these different tests can be found in the exponential relation between tolerance time and temperature (see^13,15,16^ and “Discussion” below). Thus, a linear relation between log_10_ (tolerance time) and temperature in ectothermic organisms has been known for more than a century^6,13,14,16,21–26^. This analysis of thermal tolerance has also recently been revisited as a “thermal tolerance landscape” (TTL)^15,20^. A TTL describes the temperature–time interaction of tolerance, along with information of percentwise population mortality level^20^, and thus for each mortality level (for example 50%) there is a specific temperature–time interaction, which is termed a thermal death time (TDT) curve^6^. Accordingly, multiple TDT curves which describes different mortality levels can be joined to form the thermal tolerance landscape for a population^20^. In this study, we expand this TDT curve analysis further and present a theoretical and mathematical framework that allows researchers to directly compare thermal tolerance measurements obtained during constant and dynamic experiments. Specifically, this model (and associated *R*-scripts) allows researchers to convert assessments of tolerance from static to dynamic assays (and vice versa) and to use lab measurements of tolerance to assess the severity of thermal stress experienced under temperature fluctuations.

## Theoretical foundation

**Figure 1:**
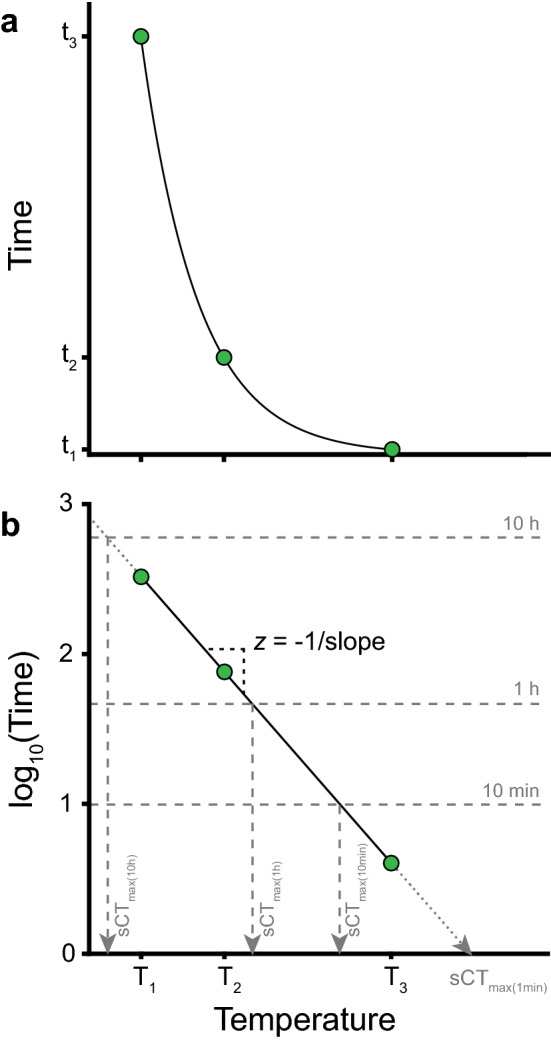
Exponential relation between temperature and t_coma_ presented as a thermal death time (TDT) curve. (**A**) Time to coma (t_coma_) decreases exponentially with temperature. (**B**) Observations from (A) presented as a TDT curve (however with coma rather than death time) where the linearity of log_10_-transformed t_coma_ versus temperature indicates the exponential relation (full line). The negative inverse slope of the TDT curve is used as the thermal sensitivity parameter *z* (*z* = the change in temperature required to change t_coma_ by an order of magnitude). Intersections between the TDT curve and horizontal lines show the temperature (sCT_max_) at which coma is expected to occur after a fixed time (e.g. 1 h, sCT_max (1 h)_). Extrapolations in either end of the TDT curve (dotted lines) are tempting but should be met with caution as the relationship is only linear in a certain time–temperature interval (see “Discussion” section).

Assessment of thermal tolerance uses various endpoints (loss of righting response, onset of spasms, coma, death^27,28^), but for a given endpoint used, the estimate of tolerance will depend critically on the duration and intensity of stress exposure. The thermal death time curve usually employs death as the tolerance assessment endpoint (as per the name), but here we use the time of coma onset, a different endpoint that is however closely related to mortality at high temperatures^29–31^. The model is likely also applicable to other endpoints and to measures of cold tolerance. In static measurements, thermal tolerance can be recorded as the duration until onset of coma (t_coma_) (Fig. 1A). The TDT curve describes thermal tolerance of a species/population using the slope of the relation between assay temperature and log_10_(t_coma_) and a point on the line (here we use sCT_max (1 h)_ which is the temperature causing heat coma after a 1-h exposure) (Fig. 1B). The slope represents a thermal sensitivity factor that describes the temperature change resulting in a one order of magnitude change in t_coma_. To be consistent with TDT curve terminology the slope is described by the parameter *z* where *z* = − 1/slope (cf.^20^), which is analogous to Q_10_ where Q_10_ = 10^10/z^. The TDT parameters from the linear regression can be used to estimate the exposure duration (t_coma_) tolerated at a specific temperature or to calculate the maximal static temperature (sCT_max_) that can be tolerated for a specific duration (but see below and associated *R*-script for details).

**Figure 2:**
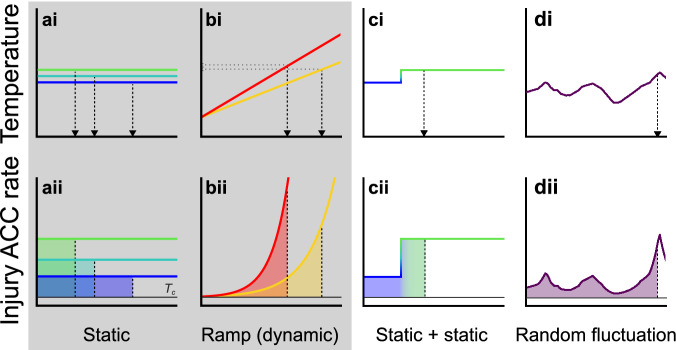
Temperature profiles of heat tolerance experiments and their corresponding temperature-dependent injury accumulation (ACC) rates based on parameters from the thermal death time curve. x-axes represent a time scale. Shaded areas under the curves represent identical amounts of accumulated injury that result in heat failure (lethal dose, **Aii**–**Dii**). The exposure duration resulting in accumulation of the lethal dose is reported as t_coma_ (static) or CT_max_ (dynamic), marked by vertical and horizontal arrows, respectively, in Ai-Di. The grey box highlights the two classical assay types (static and dynamic). Note that injury only accumulates at temperatures above a specific critical temperature (T_c_, thin black line). (**Ai**–**Aii**) Exposure to a static temperature yields a constant temperature-dependent injury accumulation rate. (**Bi**–**Bii**) Exposure to a dynamic ramp where temperature changes by a fixed rate resulting in an exponentially increasing injury accumulation rate with time. The fast ramping rate (red) results in a shorter exposure before CT_max_ is observed than the slow rate (yellow), but as the injury accumulation rate increases more slowly for the low ramping rate, the final CT_max_ is lower. (**Ci**–**Cii**) During initial exposure to a static temperature (blue), some injury is accumulated, but not sufficient to trigger coma onset. Successive exposure to another static temperature (green) produces the critical amount of injury, but as some injury had already accumulated, the exposure to the second temperature is shorter than if only this temperature had been used (compare with Aii). The prediction of the model will only hold true if injury is additive at the two static temperatures. (**Di**–**Dii**) Varying (natural or laboratory generated) temperature changes that cannot be described by a simple fixed ramping rate. However, knowing the temperature–time profile permits calculations of the injury accumulation at any time and thus estimation of t_coma_.

Another perspective of TDT curve analysis is to consider temperature as the determinant of an injury accumulation rate (Fig. 2Aii)^13–17,23^. From this perspective injury accumulation rate increases exponentially with temperature and the endpoint is then reached when the animal has accumulated the critical amount of injury (100%), illustrated graphically as the area under the curve in Fig. 2. The use of coma onset as the tolerance endpoint means that this “injury accumulation rate” is actually a “rate of failure induction”, but since heat coma and mortality is closely associated in time, we have opted to use the term injury accumulation rate. Applying the concept of injury accumulation rates, it is possible to use TDT parameters to find the tolerance limits using other combinations of static, dynamic or fluctuating stress exposure. To validate this model empirically, we use *Drosophila melanogaster* to parameterize a TDT curve with static experiments and then use the TDT parameters to predict (and test) the accumulated injury from two sequential stress exposures at two different temperatures (as in Fig. 2C) and to predict (and test) heat failure in flies exposed to randomly fluctuating temperatures (as in Fig. 2D).

To make this method accessible to researchers we describe the mathematical foundation and provide *R*-scripts to directly derive TDT parameters and use them to assess tolerance limits. The scripts can derive TDT parameters from static data sets (where time to failure, *t*_*coma*_, is measured at different constant temperatures), or from dynamic data sets (where the maximal temperature tolerated, *CT*_*max*_, is measured using different ramping rates). The scripts also allow for construction of a TDT curve using only a single static or dynamic estimate of heat tolerance, but we caution that the model will be extremely sensitive to the (untested) assumed value of *z* that must be provided. Irrespective of the type of input data, the scripts can predict heat failure at various static and dynamic measurements from TDT parameters or integrate TDT parameters with data of temperature fluctuations to predict heat stress under fluctuating conditions. To test the applicability of the model we compare estimates of heat tolerance in nine ectotherm species from many different literature sources with the assumption that each of these species are characterised by a reasonably constant species-specific heat tolerance. Specifically, we use the original experimental data to generate TDT parameters and then estimate the temperature required to cause heat failure in 1 h.

As with most models in biology the quality of the input data determines the quality of the model output. Furthermore, all models have boundaries and limitations for their applications. We therefore discuss important considerations including: (i) The importance of using experimental data for model parameterization within the time and temperature domain of interest, (ii) The risks of model extrapolation, and (iii) The need to consider model boundaries and to consider interactions with repair and hardening. These different considerations are important as they set the boundaries in time and temperature for the applicability of the model.

## Materials and methods

### Mathematical foundation

If we assume that the acute, temperature-related injury accumulation rate of an animal depends on temperature by some function *R*(*T*) then the amount of accumulated injury/damage (*d*) at some time *t* is given by:

1 $$ {\text{d}}({\text{t}}) = \mathop \smallint \limits_{{{\text{t}}_{{\text{c}}} }}^{{\text{t}}} {\text{R}}\left[ {{\text{T}}\left(\uptau \right)} \right]{\text{d}}\uptau $$

here *T*(*τ*) is the time-varying temperature regime and *t*_*c*_ is the time where injury accumulation starts. The temperature at *t*_*c*_ is *T*_*c*_, and up to this temperature the repair of injury can match the generation of injury. For Eq. (1) to hold true, however, we must require that once temperature surpasses *T*_*c*_ it will stay above *T*_*c*_. At some time *t*_*Ld*_ (the dynamic knockdown time, vertical arrow in Fig. 2Bi), a lethal dose *d*_*L*_ (area under the curve in Fig. 2Bii) has been attained and the animal is knocked down. If injury generation rate depends exponentially on temperature, and if injury repair rate is maximized at *T*_*c*_ (reached at *t*_*c*_), then the accumulated lethal dose is related to the knockdown time *t*_*Ld*_ by:

2 $$ {\text{d}}_{{\text{L}}} = {\text{R}}_{0} \mathop \smallint \limits_{{{\text{t}}_{{\text{c}}} }}^{{{\text{t}}_{{{\text{Ld}}}} }} \left( {{\text{e}}^{{{\text{k}}\left( {{\text{T}}\left( {\text{t}} \right) - {\text{T}}_{{\text{c}}} } \right)}} - 1} \right){\text{dt}} $$

Due to the potent (high *k*) relation between injury accumulation rate and temperature, and because the true *T*_*c*_ for any species is rarely known, we simplified this to (see Fig. S1 for further justification):

3 $$ {\text{d}}_{{\text{L}}} \approx {\text{R}}_{0} \mathop \smallint \limits_{{{\text{t}}_{{{\text{c}}*}} }}^{{{\text{t}}_{{{\text{Ld}}}} }} e^{{{\text{k}}\left( {{\text{T}}\left( {\text{t}} \right) - {\text{T}}_{{{\text{c}}*}} } \right)}} {\text{dt}} $$

where *t*_c*_ is some start time for integration where the temperature *T* attains some convenient value *T*_c*_ (e.g. rearing temperature) below the true *T*_*c*_. In the event that exposure temperature is constant (and above *T*_c*_) we get:

4 $$ {\text{d}}_{{\text{L}}} = {\text{t}}_{{{\text{Ls}}}} {\text{R}}_{0} {\text{e}}^{{{\text{k}}({\text{T}} - {\text{T}}_{{{\text{c}}*}} )}} $$

where t_Ls_ is the static knockdown time. Rearranging this leads to an exponential relation between the static knockdown time and temperature, which is here called sCT_max_ (static CT_max_):

5 $$ {\text{t}}_{{{\text{Ls}}}} = \upalpha {\text{e}}^{{ - {\text{k}}({\text{sCT}}_{\max } - {\text{T}}_{{{\text{c}}*}} )}} $$

where *α* = *d*_*L*_*/R*_*0*_. This is identical to the usual form of the TDT curve if *log(α)* = *sCT*_*max*_*/z* and *k* = *ln(10)/z*.

Assuming that the accumulated injury is always the same at knockdown then, once knowing knockdown time and temperature in one experimental setting, will allow calculation of expected knockdown temperatures and/or times in other experimental settings. Thus, in the case of a linearly increasing temperature ramp of the form *T(t)* = *T*_*0*_ + *bˑt*, where *T*_*0*_ is the ramp start temperature and *b* is the ramping rate, the dynamic knockdown time and temperatures can be calculated by: (– note that in the original paper^15^ the “*ln*” and the *k* in front of *(T*_c*_-T_*0*_*)* was lost in typesetting):

6 $$ {\text{t}}_{{{\text{Ld}}}} = \frac{1}{{{\text{kb}}}}\ln \left[ {{\text{kbt}}_{{{\text{Ls}}}} {\text{e}}^{{{\text{k}}\left( {{\text{sCT}}_{\max } - {\text{T}}_{0} } \right)}} + {\text{e}}^{{{\text{k}}({\text{T}}_{{{\text{c}}*}} - {\text{T}}_{0} )}} } \right] $$

7a $$ {\text{dCT}}_{\max } = {\text{T}}_{0} + \frac{1}{{\text{k}}}\ln \left[ {{\text{kbt}}_{{{\text{Ls}}}} {\text{e}}^{{{\text{k}}\left( {{\text{sCT}}_{\max } - {\text{T}}_{0} } \right)}} + {\text{e}}^{{{\text{k}}({\text{T}}_{{{\text{c}}*}} - {\text{T}}_{0} )}} } \right]. $$

One can also express *sCT*_*max*_ as a function of *dCT*_*max*_:

7b $$ {\text{sCT}}_{\max } = {\text{T}}_{0} + \frac{1}{{\text{k}}}\ln \left[ {\frac{1}{{{\text{kbt}}_{{{\text{Ls}}}} }}\left( {{\text{e}}^{{{\text{k}}\left( {{\text{dCT}}_{\max } - {\text{T}}_{0} } \right)}} - {\text{e}}^{{{\text{k}}({\text{T}}_{{\text{c}}*} - {\text{T}}_{0} )}} } \right)} \right]. $$

If the temperature varies randomly (and therefore is not monotonically increasing) it is no longer possible to calculate an expected knockdown temperature from knockdown temperatures obtained in static or dynamic ramp experiments. However, the expected knockdown time can still be found by evaluating (usually numerically) the integral of Eq. (3) as a function of time.

#### Using the equations

We provide two *R-*scripts (https://github.com/MOersted/Thermal-tolerances, see guide in Supplementary Information) to aid TDT parameterization from static and dynamic assays. One script establishes the TDT parameters from the knockdown time at two or more static temperatures (Fig. 1), while the other script derives TDT parameters from two or more values of *dCT*_*max*_ obtained by different ramping rates and/or start temperatures (Fig. 2B). Using dynamic input data, *sCT*_*max*_ and *z* is estimated either through nonlinear curve fitting in case *dCT*_*max*_ is available for three or more ramping rates, or by solving two equations (either 7a or 7b) with two unknowns when *dCT*_*max*_ is known for two ramping rates (see Supplementary Information for details).

Knowing only one dCT_max_ or sCT_max_ will not suffice to establish a TDT curve since *k* (and thus *z*) in Eqs. (7a) and (7b) will be left unknown. In such cases, an estimated value of *z* must be supplied (some guidance is provided in Supplementary Information, Table S1), to allow the establishment of a crude TDT curve, which will be subject to uncertainty and sensitive to extrapolation (see “Discussion” section).

Once the TDT curve is parameterized, both scripts have four available outputs; (i) estimated exposure duration (t_coma_) tolerated at specific temperatures, (ii) estimated static temperature (*sCT*_*max*_) that can be tolerated for specific durations, (iii) estimated maximal dynamic temperature *dCT*_*max*_ that can be tolerated in experiments with specific ramp rates, and (iv) estimated injury accumulation under fluctuating temperatures (Fig. 2D), and thus the exposure duration tolerated before a given percent of lethal injury has accumulated.

### Empirical experiments for model validation

#### Animal husbandry

*Drosophila melanogaster* (collected in Denmark, 2011) were kept in bottles with oat-based Leeds medium^11^ (19 °C, constant light). Experimental flies were produced by transferring a spoonful media containing egg/larvae to fresh bottles (23 °C, 14:10 L:D cycle). Emerging flies were collected every two days and after 2–5 days flies were briefly anaesthetised with CO_2_ (< 5 min), then separated by sex and returned to fresh vials. Flies were allowed > 2 days to recover from anaesthesia before experiments^32^, resulting in 4–8 days old experimental flies.

#### Thermal death time (TDT) curves

TDT curves were generated by exposing flies to different stressful static temperatures and measuring the time to coma (t_coma_, like Fig. 1). Experiments were conducted at temperatures resulting in heat coma onset ranging from ~ 10 min to 5 h using the experimental setup described in^15^. Briefly, flies were placed individually in 5-mL glass vials with a droplet of Leeds medium in the cap (food and water source), then mounted to a rack and submerged in a preheated temperature-controlled water bath. The median value of t_coma_ was reported for each temperature allowing for long experiments (> 30 min) to be terminated once more than half of the flies had entered heat coma (See^15^ for use of median values). A TDT curve was parameterized for each sex by linear regression of the log_10_-transformed median t_coma_ against the assay temperature as described above (Fig. 1).

#### Test of injury additivity between two static temperatures

To investigate whether heat injury acquired at different temperatures is additive, flies were exposed to one stressful temperature (T_1_) and successively transferred to another stressful temperature (T_2_) (Fig. 2C). The two temperatures were selected from the TDT curve: a ‘low’ temperature (~ 36.5 °C) with expected t_coma_ > 4 h and a ‘high’ temperature (~ 39.5 °C) with expected t_coma_ < 1 h. Experiments were performed with both the ‘high’ and ‘low’ temperature as T_1_ to examine whether additivity was independent of the heat stress intensity during the first exposure. In each experimental run, six groups (n = 10) of both sexes were placed at T_1_ and then transferred to T_2_ at regular intervals for recording of t_coma_. For each combination of sex and treatment order (‘high’ or ‘low’ temperature as T_1_) the median exposure time at T_1_ and T_2_ was recalculated as fractions of the t_coma_ predicted by the TDT curve. As an example, an initial exposure of 15 min to the higher test temperature (39.5 °C, TDT curve-predicted t_coma_ = 50 min) corresponds to a fraction of 15 min/50 min = 0.3 of injury resulting in coma. The hypothesis of additivity predicts that the fractions of accumulated injury resulting in coma should sum to 1, and accordingly the predicted fraction of time to coma at T_2_ should be 0.7. Thus, if the lower test temperature (36.5 °C) has a TDT curve-predicted median t_coma_ of 300 min, a fraction of 0.7 corresponds to 300 min⋅0.7 = 210 min.

#### Test of injury additivity during temperature fluctuations

Animals in the field experience temperature fluctuations. If injury is additive, it should be possible to predict t_coma_ under such conditions by considering fluctuations as many additive exposures to different temperatures each characterised by an injury accumulation rate (calculated from TDT curve, Fig. 2D). To investigate whether heat injury during fluctuating temperatures can be modelled from TDT parameters, t_coma_ was observed in flies exposed to randomly fluctuating temperatures and compared to a TDT curve-predicted t_coma_. Flies were divided into 13 experimental groups (n = 16–18) for each sex. Groups were introduced to the water bath at different times, which was randomly and sequentially programmed to heat or cool. Consequently, each group experienced a unique temperature profile (range: 34.5–42.5 °C). The median t_coma_ for each sex and experimental group was compared to the t_coma_ predicted by the TDT curve. Specifically, to predict t_coma_ under these conditions the associated *R*-script was given the average temperature for each 10-s period and injury accumulation for each period was then calculated from the TDT curve. Injury accumulated during each 10-s interval was summed until the critical amount of injury had accumulated resulting in predicted t_coma_.

### Model validation using literature data

Measures of heat tolerance from different publications are difficult to compare as they are based on different experimental procedures or conditions. To examine whether our model can improve comparison of published tolerance estimates, we collected heat tolerance measures from both static and dynamic assays for nine ectothermic species including a crustacean (*Daphnia magna* Straus 1820), insects (*D. melanogaster* Meigen 1830, *Drosophila subobscura* Collin 1936, *Glossina pallidipes* Austen 1903, *Tenebrio molitor* L. 1758), collembolans (*Folsomia candida* Willem 1902, *Orchesella cincta* L. 1758) and fishes (*Gambusia affinis* Baird & Girard 1853, *Salmo salar* L. 1758). Literature search was aided by an overview of dynamic assays with at least three ramping rates^33^, the GlobTherm database^34^ and a review of CT_max_ in aquatic ectotherms^35^. For static assays the time of failure and assay temperature were recorded while the ramping rate, CT_max_ and the initial temperature (t_0_) were noted for dynamic assays. Acclimation temperature was recorded if specified. The aim was to gather multiple heat tolerance measures from the same publication to provide input for TDT curves, but publications with fewer assay temperatures or ramping rates were also included to examine how the model operated using assumed values of *z*. The data set included 155 static entries and 227 dynamic entries from 55 publications. For all studies included the associated *R*-scripts were used to derive TDT parameters to estimate the static temperature that causes heat failure after a 1-h exposure (sCT_max(1 h)_). For studies with single tolerance measures we assumed the mean species value of *z* calculated from the other publications in our analysis (Table S1).

## Results

### Thermal death time curves

**Figure 3:**
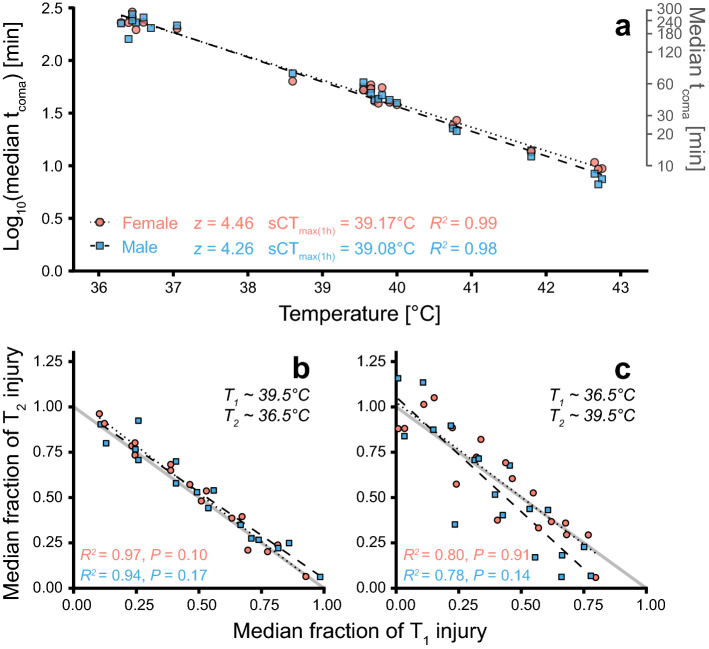
Thermal death time curves and test of injury additivity between two temperatures. (**A**) Flies were exposed to a static stressful temperature until coma onset (t_coma_). Each point is the median t_coma_ of a group (n = 2–10) and TDT curves are made for each sex by linear regressions of median log_10_(t_coma_) vs. assay temperature. (**B**) Groups of flies (n = 5–6 per sex) were exposed to a high temperature (~ 39.5 °C) and then, after different durations, transferred to a lower temperature (~ 36.5 °C) where median t_coma_ was recorded. The exposures are shown as fractions of injury resulting in coma at T_1_ (x-axis) and T_2_ (y-axis), where small and large fractions indicate brief and long exposures, respectively (i.e. brief exposure results in small accumulation of injury relative to the critical amount of injury). If injury acquired at two different temperatures is additive, the two fractions should sum to 1, i.e. follow the (full) line of additivity. (**C**) A similar experiment as (**B**), but with initial exposure to ~ 36.5 °C and the subsequent exposure to ~ 39.5 °C.

Median time to heat coma (t_coma_) ranged from 7 to 289 min (~ 5 h) in flies exposed to constant, stressful temperatures ranging 36.30–42.75 °C (Fig. 3A). The resulting TDT curves were well-fitted (*R*^2^ > 0.98) for both sexes, showing that the exponential function is appropriate to describe the negative relation between temperature and t_coma_. These TDT parameters (*z* and sCT_max(1 h)_) were used to characterise temperature-specific injury accumulation rates in subsequent experiments.

### Test of injury additivity between two static temperatures

To investigate whether heat injury acquired at two stressful temperatures is additive, groups of flies were exposed to a static temperature (T_1_) and subsequently exposed to another static temperature (T_2_) (Fig. 3B,C). Injury accumulated at the two temperatures was scored as the fraction of injury required to enter coma at T_1_ and T_2_, respectively (calculated from TDT curves, Fig. 3A), and these fractions summed to ~ 1 (graphically points are scattered around the line of additivity, *R*^2^ > 0.78, Fig. 3B,C). This indicates that heat injury at two static stressful temperatures is additive, which is supported statistically as the linear regressions of the fractional injury accumulation to t_coma_ at T_1_ *vs*. T_2_ were not significantly different from the line of additivity for neither sex nor temperature sequence (F_(2,14–16)_ < 2.7, *P* > 0.1, Fig. 3B,C, Table S2).

### Test of injury additivity during random temperature fluctuations

**Figure 4:**
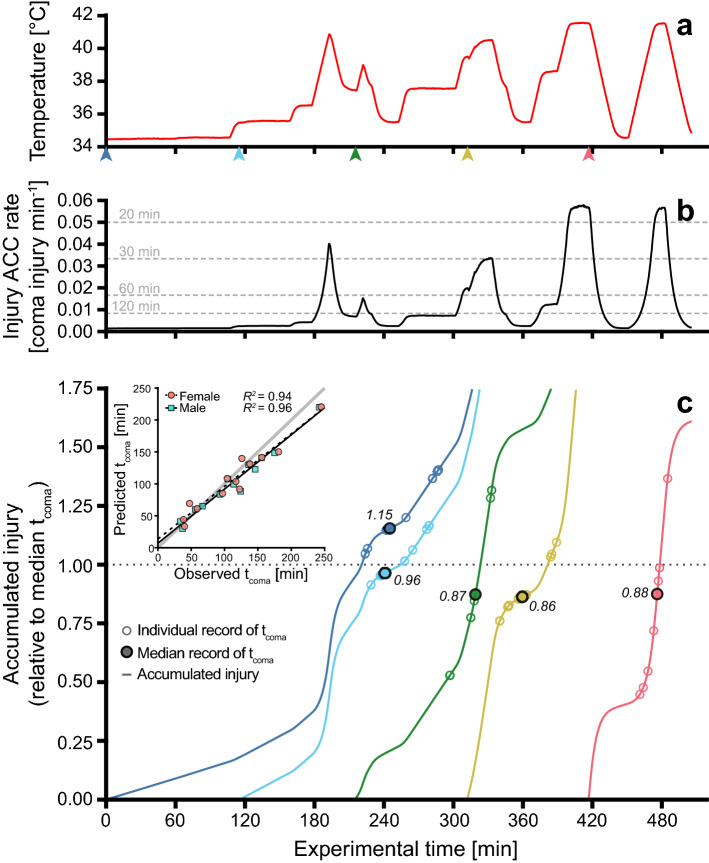
Test of injury additivity during random temperature fluctuations. (**A**) Fluctuating temperature profile from one of the experiments. (**B**) The corresponding temperature specific injury accumulation (ACC) rates for each 10-s interval calculated from the TDT curve (Fig. 3A). Rates are presented as a fraction of coma injury min^−1^ (i.e. 0.01 corresponds to an injury rate that would cause coma in 100 min), and dashed lines show the injury accumulation rate that, if held constant, would result in coma after 20, 30, 60 and 120 min. (**C**) Groups of flies (n = 8–9) were introduced at different times during the assay (arrowheads in (**A**)), and thus each group experienced a unique sequence of fluctuating temperatures. The temperature-specific injury accumulation rates were used to predict the gradual injury accumulation (coloured lines), and accordingly, *when* each experimental group should have accumulated the critical amount of injury according to the TDT curve (fraction = 1, dotted line). Numbers accompanying median points are the calculated amount of injury accumulated at that specific time for this group. (Inset) Observed vs predicted t_coma_ from all experiments (grey line of unity).

Groups of flies were exposed to randomly fluctuating temperatures to investigate whether injury accumulation was also additive under these conditions (Figs. 2D, 4A). Each group experienced a unique sequence of stressful temperatures resulting in exposures spanning 33–245 min before the last fly entered coma. For each of the random temperature exposures the accumulated amount of injury was predicted using TDT parameters and the accompanying *R*-script (Fig. 4B). Median t_coma_ was recorded for each sex in the 13 test groups and compared to the predicted t_coma_ for each specific fluctuating temperature sequence (Fig. 4C). Predicted and observed t_coma_ correlated strongly in both sexes (*R*^2^ > 0.94), and though the linear regressions deviated slightly from the line of unity (F_(2,11)_ > 5.9; *P* < 0.02, Table S3), the predicted and observed t_coma_ were not significantly different (inset in Fig. 4C, *Mann–Whitney U test*, male: W = 96, *P* = 0.58; female: W = 88, *P* = 0.84). The model calculated the mean amount of injury accumulated at the median observed t_coma_ to be 1.10 (range: 0.75–1.36) and 1.09 (range: 0.86–1.46) for males and females, respectively, where 1.00 is the TDT curve predicted amount causing coma (Fig. 4C).

### Using TDT parameters to compare heat tolerance measures from the literature

**Figure 5:**
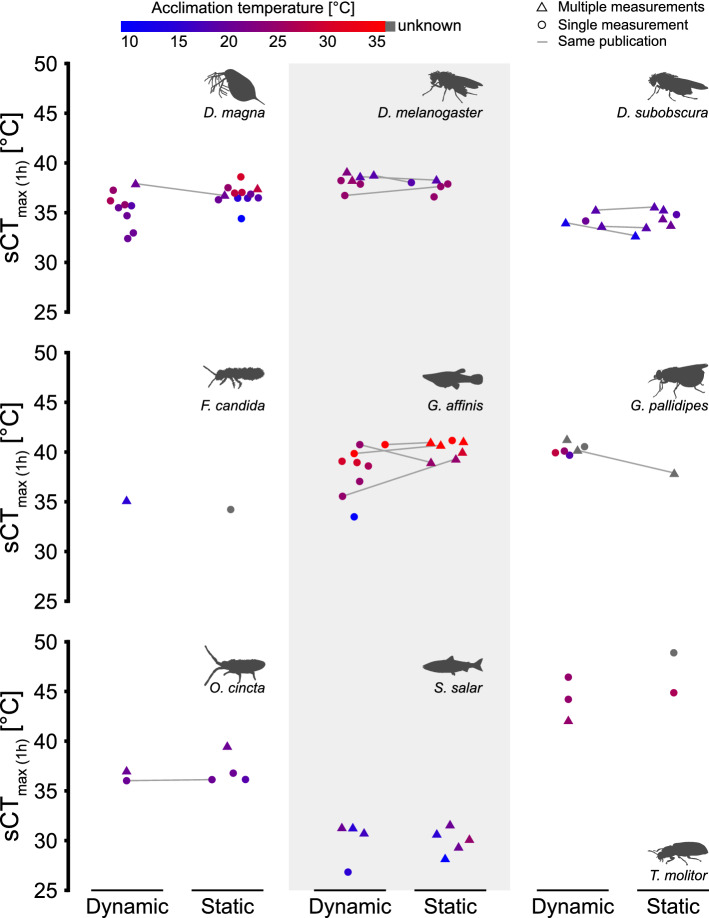
Heat tolerance measures from the literature re-calculated to the temperature resulting in heat failure after a 1-h exposure (sCT_max (1 h)_). Heat tolerance measurements from both dynamic and static assays were obtained from the literature for nine ectothermic species and used as input for the associated *R*-scripts. If multiple measures of the same assay type were available in a publication (triangles), these measurements were used for TDT curve parameterization and then calculate sCT_max (1 h)_. If only a single measurement was available (circle) an estimated value of *z* was supplied to create a TDT curve for calculation of sCT_max (1 h)_ (see main text). Connected points represent publications that provided both dynamic and static measures and colour indicates acclimation temperature.

Literature values of heat tolerance were obtained for nine ectotherm species and for each publication we used values as model input to calculate a common heat tolerance estimate; the static temperature estimated to result in heat failure after a 1-h exposure (sCT_max (1 h)_, Fig. 5). Input data included both dynamic and static experiments and after conversion to a common metric sCT_max (1 h)_ we generally found overlap within species and it is for example possible to discern large interspecific differences in heat tolerance (compare *D. melanogaster* and *D. subobscura* or *G. affinis* and *S. salar*, Fig. 5). In species with a relatively large intraspecific variation, higher acclimation temperatures tend to increase heat tolerance, although this was not formally tested.

## Discussion

### Fitting tolerance time versus temperature to build a thermal death time curve

The high coefficients of determination found in the *D. melanogaster* TDT curves (Fig. 3A) are not uncommon and the exponential relation has consistently been found to provide a good fit of tolerance time vs. temperature in ectotherms^3,15,20,22–24^. Tolerance time vs. temperature data are also well fitted to Arrhenius plots which are based on thermodynamic principles (see for example^15,36^) and the absence of breakpoints in such plots provides a strong indication (but not direct proof) that the cause of coma/heat failure under the different intensities of acute heat stress is related to the same physiological process regardless whether failure occurs after 10 min or 10 h^2,3^ (but see “Discussion” section below). Despite the superior theoretical basis of Arrhenius analysis, we proceed with simple linear regressions of log_10_-transformed *t*_*coma*_ (TDT curve) as this analysis likewise provides a high *R*^2^ and is mathematically more straightforward. The physiological cause(s) of ectotherm heat failure are poorly understood^37,38^ but we argue that they are founded in a common process where heat injury accumulates at a temperature-dependent rate until a species-specific critical dose is attained (area below the curve and above T_c_ in Fig. 2). Thus, the organism has a fixed amount (dose) of thermally induced stress that it can tolerate before evoking the chosen endpoint. The experienced temperature of the animals then dictates the rate of which this stress is acquired, and accordingly when the endpoint is reached (Fig. 2) It is this reasoning that leads to TDT curves and explains why heat stress can be additive and thus also determines the boundaries of TDT curve modelling.

### Injury is additive across different stressful assay temperatures

If heat stress acquired at intense and moderate stress within the span of the TDT curve acts through the same physiological mechanisms or converges to result in the same form of injury, then it is expected that injury is additive at different heat stress intensities. This hypothesis was tested by exposing flies sequentially to two static temperatures (different injury accumulation rates) and observe whether coma occurred as predicted from the summed injury (Fig. 2C). The accumulated heat injury at the two temperatures was found to be additive regardless of the order of temperature exposure (Fig. 3B,C). This finding is consistent with a conceptually similar study using speckled trout which also found strong support for additivity of heat stress at different stressful temperatures^13^. The exact physiological mechanism of heat injury accumulation is interesting to understand in this perspective, but it is not critical as long as the relation between temperature and injury accumulation rate is known.

**Figure 6:**
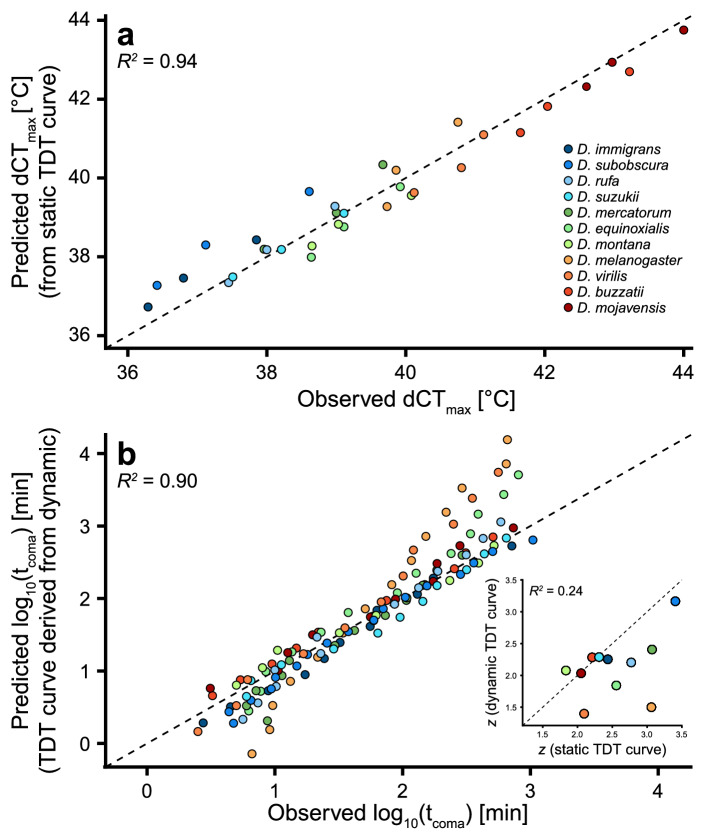
Conversion of heat tolerance measures between static and dynamic assays in *Drosophila*. Data from^43^. (**A**) Heat tolerance (dCT_max_, *d* for dynamic assays) plotted against predicted dCT_max_ derived from species-specific TDT curves created from multiple (9–17) static assays. Data are presented for three different ramping rates (0.05, 0.1 and 0.25 °C min^-1^). Note that this graph is adapted from Fig. 4b in^15^. (**B**) TDT parameters based on dCT_max_ from three dynamic tests were used to predict t_coma_ in static assays. Each point represents an observed vs. predicted value of species- and temperature-specific log_10_(t_coma_). (Inset) Species values of the thermal sensitivity parameter *z* parameterized from TDT curves based on static assays (x-axis) or dynamic assays (y-axis). The dashed line represents the line of unity in all three panels.

If injury accumulation is additive irrespective of the order of the heat exposure, we can extend the model to fluctuating temperature conditions. We have previously done this by accurately predicting dynamic CT_max_ from TDT parameters obtained from static assays for 11 *Drosophila* species (Fig. 6A, see “Discussion” section below and^15^). Here we extend this to temperature fluctuations that cannot be described by a simple mathematical ramp function. Specifically, groups of flies were subjected to randomly fluctuating temperatures and the observed t_coma_ was then compared to t_coma_ predicted using integration of heat injury based on TDT parameters (Fig. 4). The injury accumulation (Fig. 4C) was calculated by introducing the fluctuating temperature profiles in the associated *R*-script and the observed and predicted t_coma_ was found to correlate well (*R*^2^ > 0.94) across the 13 groups tested for each sex. These results further support the idea that injury is additive across a range of fluctuating and stressful temperatures and hence that similar physiological perturbations are in play during moderate and intense heat stress. It is important to note that in these experiments, temperatures fluctuated between 34.5 and 42.5 °C and accordingly the flies were never exposed to benign temperatures that could allow repair or hardening (see below).

In conclusion, empirical data (present study;^6,13,14,22^) support the application of TDT curves to assess heat injury accumulation under fluctuating temperature conditions both in the lab and field for vertebrate and invertebrate ectotherms. Potential applications could be assessment of injury during foraging in extreme and fluctuating environments (e.g. ants in the desert^39^ or lizards in exposed habitat^40^) or for other animals experiencing extreme conditions^41,42^. The associated *R*-scripts allow assessment of percent lethal damage under such conditions if the model is provided with TDT parameters and information of temperature fluctuations (but see “Discussion” section of model limitations below).

### Model application for comparison of static versus dynamic data

There is little consensus on the optimal protocol to assess ectotherm thermal tolerance and many different types of static or dynamic tests have been used to assess heat tolerance. TDT curves represent a mathematical and theoretical approach to reconcile different estimates of tolerance as the derived parameters can subsequently be used to assess heat injury accumulation at different rates (temperatures) and durations^13,15,16^. Here we provide *R*-scripts that enable such reconciliation and to demonstrate the ability of the TDT curves to reconcile data from static vs. dynamic assays we used published measurements of heat tolerance for 11 *Drosophila* species using three dynamic and 9–17 static measurements for each species^43^. Introducing data from only static assays we derived TDT parameters and subsequently used these to predict dynamic CT_max_ that were compared to empirically observed CT_max_ for three ramp rates (Fig. 6A). In a similar analysis, TDT parameters were derived from the three dynamic (ramp) experiments to predict t_coma_ at different static temperatures which were compared to empirical measures from static assays (Fig. 6B). Both analyses found good correlation between the predicted and observed values regardless whether the TDT curve was parameterized from static or dynamic experiments (Fig. 6). However, predictions from TDT curves based on three dynamic assays were characterised by more variation, particularly when used to assess tolerance time at very short or long durations. Furthermore, *D. melanogaster* and *D. virilis* which had the poorest correlation between predicted and observed t_coma_ in Fig. 6B had values of *z* from the TDT curves based on dynamic input data that were considerably different from values of *z* derived from TDT curves based on static assays (Fig. 6B inset). In conclusion TDT curves (and the associated *R*-scripts) are useful for conversion between static and dynamic assessment of tolerance*.* The quality of model output depends on the quality and quantity of data used as model input, and in this example the poorer model was parameterized from only three dynamic assays while the stronger model was based on 9–17 static assays (see also “Discussion” section below).

### Model application for comparison of published data

Thermal tolerance is important for defining the fundamental niche of animals^1,2,4^ and the current anthropogenic changes in climate has reinvigorated the interest in comparative physiology and ecology of thermal limits in ectotherms. Meta-analyses of ectotherm heat tolerance data have provided important physiological, ecological and evolutionary insights^5,44–46^, but such studies are often challenged with comparison of tolerance estimates obtained through very different methodologies.

Species tolerance is likely influenced by acclimation, age, sex, diet, etc.^47^ and also by the endpoint used (onset of spasms, coma, death, etc.^27^). Nevertheless, we expected heat tolerance of a species to be somewhat constrained^45^, so here we tested the model by converting literature data for nine species to a single and species-specific estimate of tolerance, sCT_max (1 h)_, the temperature that causes heat failure in 1 h (Fig. 5). The overwhelming result of this analysis is that TDT parameters are useful to convert static and dynamic heat tolerance measures to a single metric, and accordingly, the TDT model and *R*-scripts presented here have promising applications for large-scale comparative meta-analyses of ectotherm heat tolerance where a single metric allows for qualified direct comparison of results from different publications and experimental backgrounds. While this is an intriguing and powerful application, we caution that careful consideration should be put into the limitations of this model (see “Discussion” section below).

### Practical considerations and pitfalls for model interpretation

As shown above it is possible to convert and reconcile different types of heat tolerance measures using TDT parameters and these parameters can also be used to model heat stress under fluctuating field conditions. Modelling and discussion of TDT predictions beyond the boundaries of the input data has recently gained traction (see examples in^48,49^) but we caution that the potent exponential nature of the TDT curve requires careful consideration as it is both easy and enticing to misuse this model.

#### Input data

The quality of the model output is dictated by the input used for parameterization. Accordingly, we recommend TDT parameterization using several (> 5) static experiments that should cover the time and temperature interval of interest, e.g. temperatures resulting in t_coma_ spanning 10 min to 10 h, thus covering both moderate and intense heat exposure. Such an experimental series can verify TDT curve linearity and allows modelling of temperature impacts across a broad range of temperatures and stress durations^13,15,22^. It is tempting to use only brief static experiments (high temperatures) for TDT parameterization, but in such cases, we recommend that the resulting TDT curve is only used to describe heat injury accumulation under severe heat stress intensities. Thus, the thermal sensitivity factor *z* represents a very powerful exponential factor (equivalent to Q_10_ = 100 to 100,000;^15^) which should ideally be parametrized over a broad temperature range (see below). We also include a script that allows TDT parametrization from multiple ramping experiments and again we recommend a broad span of ramping rates to cover the time/temperature interval of interest. A drawback of ramping experiments is the relatively large proportion of time spent at benign temperatures where there is no appreciable heat injury accumulation. Thus, dynamic experiments can conveniently use starting temperatures that are close to the temperature where injury accumulation rate surpasses injury repair rate (see “Discussion” section of “true” T_c_ below, in Supplemental Information and^19^ for other considerations regarding ramp experiments).

A final methodological consideration relates to body-temperature in brief static experiments where the animal will spend a considerable proportion of the experiment in a state of thermal disequilibrium (i.e. it takes time to heat the animal). To avoid this, we recommend direct measurement of body temperature (large animals) or container temperature (small animals), and advise against excessive reliance on data from test temperatures that results in coma in less than 10 min.

#### Extrapolation

**Figure 7:**
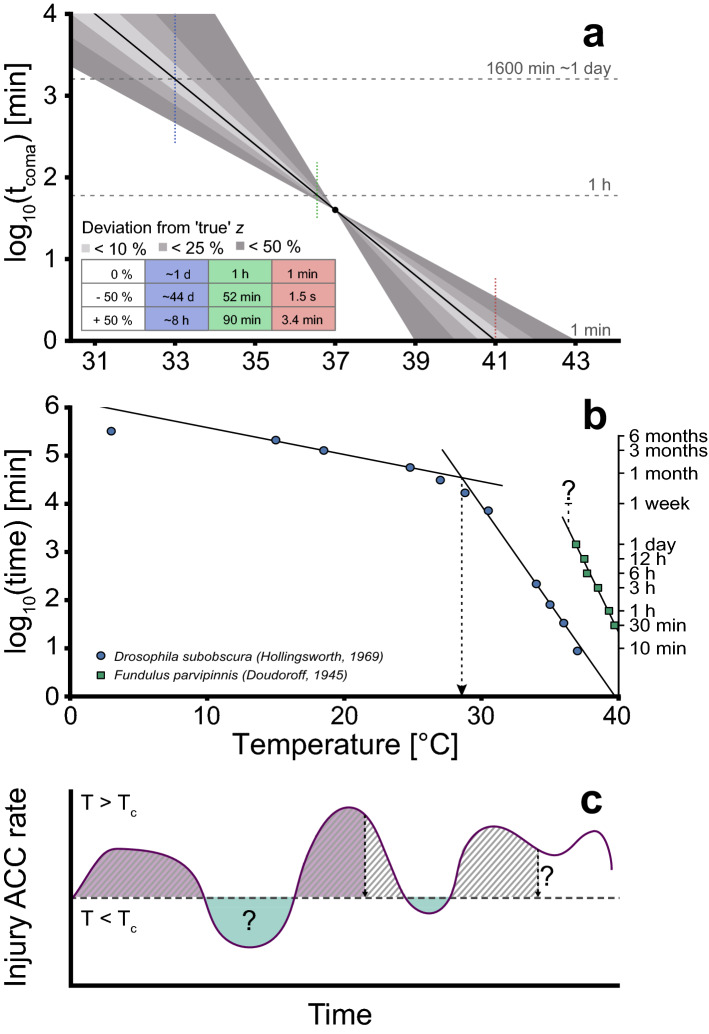
Potential pitfalls of extrapolation and the ambiguity of heat damage repair and hardening. (**A**) A theoretical TDT curve created from a single point (37 °C, 40 min) with an assumed ‘true’ value of *z* (black line). Grey areas show the TDT curve produced from the same point with deviations from the ‘true’ *z* of ± 10–50%. Horizontal lines are used to compare estimates of sCT_max_ for 1 min, 1 h and 1600 min, while the vertical coloured lines are used to compare time estimates for the temperature of the sCT_max_ for the ‘true’ TDT curve (calculated times in table) (**B**) The linearity of TDT curves should only be assumed within the time–temperature domain where it is parameterized, and it may vary in temperature and time between species. Data and TDT curve estimates for *D. subobscura*^22^ and *F. parvipinnis*^25^. The dashed line for *Fundulus* represents the temperature with no mortality within the tested time domain (≤ 1 week). The dashed arrow indicates the breakpoint temperature found by^22^. (**C**) Hypothetical fluctuating temperature profile where temperature (and accordingly the injury accumulation (ACC) rate) fluctuate around the incipient lethal temperature *T*_*c*_ (the temperature where injury accumulation rate surpasses injury repair rate, i.e. net injury accumulation). The purple area indicates the part of the temperature profile that would attain the critical amount of injury, under the assumption that no repair or hardening (i.e. processes counteracting injury accumulation) takes place in the green shaded areas. However, when little is known about the processes counteracting injury accumulation and their relation to temperature, it is difficult to predict when coma onset occurs (hatched area).

Most studies of ectotherm heat tolerance include only a single measure of heat tolerance which is inadequate to parameterize a TDT curve. However, a TDT curve can still be generated from a single measure of tolerance (static or dynamic) if a value of *z* is assumed (see Supplemental Information). As *z* differs within species and between phylogenetic groups (Table S1^15,20^), choosing the appropriate value may be difficult and discrepancies between the ‘true’ and assumed *z* represent a problem that should be approached with care. In Fig. 7A we illustrate this point in a constructed example where a single heat tolerance measurement is sampled from a ‘true’ TDT curve (full line; t_coma_ = 40 min at 37 °C). Along with this ‘true’ TDT curve we depict the consequences for model predictions if the assumed value of *z* is misestimated by ± 50%. Extrapolation from the original data point is necessary if an estimate of the temperature that causes coma after 1 h is desired, however due to limited extrapolation (from 40 to 60 min), estimation of sCT_max (1 h)_ values based on the ‘true’ and *z* ± 50% are not very different (< ± 0.22 °C in this example). Accordingly, moderate extrapolations are associated with minor latent errors and such assumptions were the basis for many data points in our comparative analysis (Fig. 5). If extensive extrapolation is used (here 40-fold from 40 min to either 1 or 1600 min, Fig. 7A), the assumed *z* results in sCT_max_ estimates varying ± 2 °C from the true value and even more dramatic discrepancies are seen if *t*_*coma*_ is calculated for the temperatures resulting in the ‘true’ sCT_max_ for 1 min or 1600 min (41 and 33 °C, respectively, table in Fig. 7A). Due to the powerful exponential nature of the TDT curve, extrapolation to 41 or 33 °C with values of *z* ± 50% gives predicted t_coma_ of 1.5 s-3.42 min (‘true’ t_coma_ = 1 min) and 8 h-44 days (‘true’ t_coma_ ~ 1 day). Accordingly, excessive extrapolation of TDT curves should be avoided as even moderate errors in the estimate of *z* can result in dramatic output errors if the TDT curve is extrapolated beyond the domain of the input data.

#### Breakpoints and incipient lethal temperature

TDT curves are established at critically high temperatures and another cause of concern for extrapolation of TDT curves relates to the boundaries of the model. Below some temperature [incipient lethal temperature *c.f.*^13^, here termed *T*_*c*_ (see “Mathematical foundation” section], the processes related to acute heat injury will no longer determine the duration of survival, and graphically this is represented as a breakpoint on the TDT curve (analogous to an Arrhenius breakpoint) (Fig. 7B). The premise of the TDT curve is not valid below *T*_*c*_ and hence other processes will limit survival below this temperature resulting in a breakpoint. For most species *T*_*c*_ is unknown, and it is possible that this value (breakpoint) will also depend on other factors (acclimation, age, sex, diet, etc.)(compare *Fundulus* and *Drosophila*, Fig. 7B). Accordingly, extrapolation beyond the parameterized time–temperature domain of the TDT curve should be met with great caution for instance when modelling over diurnal temperature fluctuations as this likely includes temperatures below the incipient temperature.

#### The role of repair and acclimation

From the present study and historical data^13,14,16,22,25^ it is clear that damage attained within the boundaries of the TDT curve is additive and that this model can be used to assess heat injury accumulation during fluctuations. Additivity is, however, only empirically validated within the boundaries of the TDT curve (i.e. above *T*_*c*_; Fig. 7B), and at temperatures below *T*_*c*_ it is likely that heat injury can be repaired (Fig. 7C). A study using split-dose heat exposures interspaced by benign temperature exposure found that breaks (> 6 h) between heat exposure disrupted additivity, suggesting that injury is repaired at benign temperature^50^. Injury repair rate is largely understudied but repair rate is generally increasing with temperature^51–53^. It is therefore an intriguing and promising idea to include a temperature-dependent repair function in more advanced modelling of heat injury. Until such repair processes are introduced in the model, we recommend that additivity of heat injury is evaluated critically if it involves periods at temperatures both above and below *T*_*c*_ (i.e. over consecutive days, see also^13^). An alternative, but not mutually excluding, explanation of increased heat resilience in split-dose experiments relates to the contribution of heat hardening as it is likely that the first heat exposure in a series can induce hardening responses that increase resilience (and thus change the TDT parameters) when a second heat exposure occurs. Such issues of repeated thermal stress have been discussed previously^54^ but for the purpose of the present study the main conclusion is that simple TDT curve modelling is not applicable to fluctuations bracketing *T*_*c*_ unless this is empirically validated. Future studies could address this issue as inclusion of repair functions would add further promise to the use of TDT curves in modelling of the impacts of temperature fluctuations.

## Supplementary Information

Supplementary Information.
