# Supplementary material for: A unifying model to estimate thermal tolerance limits in ectotherms across static, dynamic and fluctuating exposures to thermal stress
Source: Sci Rep. 2021 Jun 18;11:12840. doi: 10.1038/s41598-021-92004-6 (PMC8213714; doi:10.1038/s41598-021-92004-6)
Supplement: Supplementary file 1 — Supplementary Information. [file 41598_2021_92004_MOESM1_ESM.pdf]

Supplementary information

A unifying model to estimate thermal tolerance limits in ectotherms across static, dynamic and fluctuating exposures to thermal stress

Lisa Bjerregaard Jørgensen, Hans Malte, Michael Ørsted, Nikolaj Andreasen Klahn & Johannes Overgaard

Zoophysiology, Department of Biology, Aarhus University, 8000 Aarhus C, Denmark

Contents

Fig. S1 2
Guide to R-scripts 4
1 TDT curve from static input data 6
1.1 Output: Tolerable temperature at a given exposure time 7
1.2 Output: Knockdown time at a given temperature 8
1.3 Output: Dynamic CT_{max} in ramping assays 9
1.4 Output: Knockdown time under randomly fluctuating temperatures 11
2 TDT curve from dynamic input data 12
2.1 Output: Tolerable temperature at a given exposure time 15
2.2 Output: Knockdown time at a given temperature 15
2.3 Output: Dynamic CT_{max} in ramping assays 16
2.4 Output: Knockdown time under randomly fluctuating temperatures 17
Table S1 18
Table S2 19
Table S3 20
Supplementary references 21

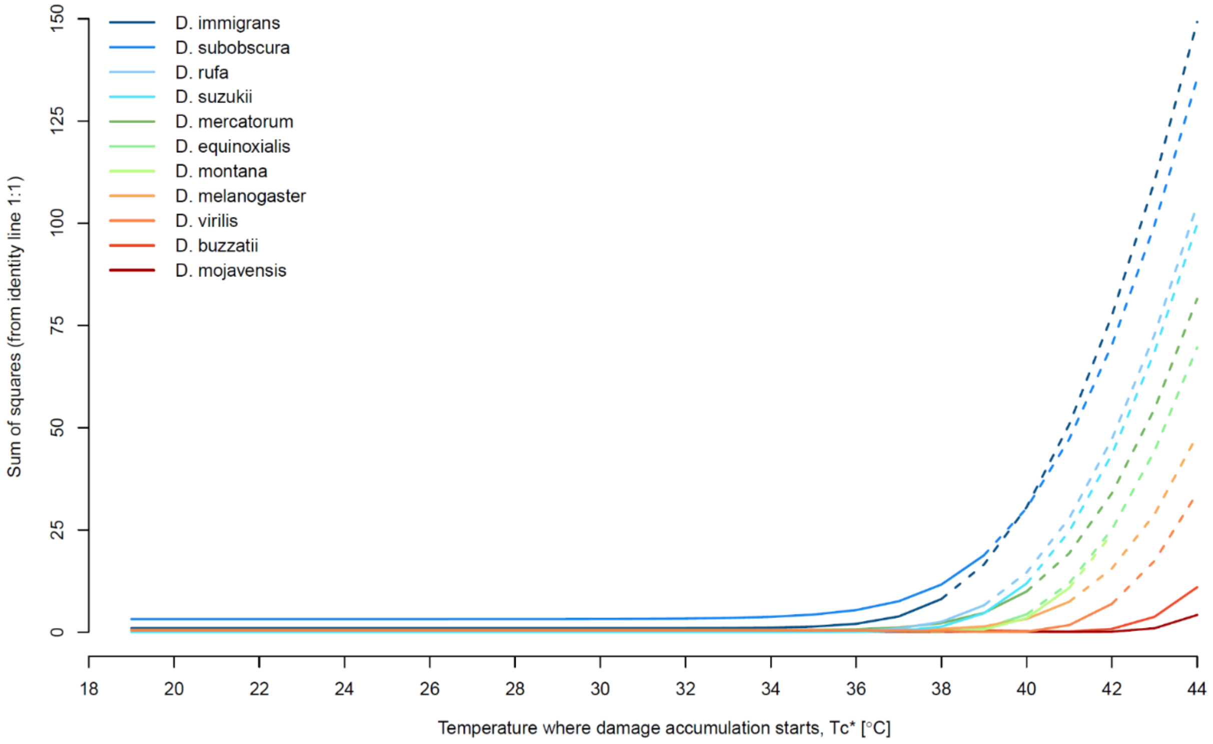

Fig. S1. T_c is the temperature above which net injury accumulation starts. As the 'true' T_c is rarely known, we recommend using some convenient value T_{c*} below the true T_c (this could for example be the rearing temperature). With data from Jørgensen et al. (2019b) where heat tolerance was measured for 11 *Drosophila* species using three dynamic and 9-17 static measurements for each species, we here evaluate the effects of changing T_{c*} on prediction accuracy when predicting dynamic CT_{max} (dCT_{max}) from TDT parameters derived from static measurements (sCT_{max}). For each of the 11 species, dCT_{max} was predicted using T_{c*} values from 19 to 44°C in 1°C increments. 19°C was chosen as this was the rearing temperature of the flies. For each T_{c*}, a linear fit of predicted dCT_{max} against empirically observed dCT_{max} at three ramp rates (0.05, 0.10, and 0.25 °C/min) was compared to the identity line (a 1:1 relationship between predictions and observations). The prediction accuracy was evaluated as the deviation (sum of squares; SS) from this identify line. Each line represents this species-specific deviation as a function of T_{c*} with a solid line until the maximum predicted dCT_{max} for each species and a dashed line thereafter. For *D. montana*, the residual SS of the model was below a precision limit at T_{c*} above 42 °C. If the chosen value of T_{c*} is above the true T_c the model becomes inadequate to model the empirical ramp experiment, because it does not include the damage accumulated from reaching the true T_c until the chosen value of a high T_{c*} and consequently, the SS of the fit between the modeled and empirical observations increases (as the model will overestimate heat tolerance by underestimating the amount of accumulated injury). The analysis shown here is not ideal to define the true T_c, but it shows that the predictive power of the model is only diminished at very high T_{c*}, often higher than the predicted dCT_{max}. Accordingly, to avoid excluding accumulated injury it is safer to use a low value of T_{c*} than a high value. In this case, even if T_{c*} is well below

the true T_c and thus allows the model to accumulate injury, the injury accumulated at the temperatures below the true T_c is negligible as the corresponding injury accumulation rate at lower temperatures is small. The other side of this argument is that ramp experiments should not be started at very high temperatures (T_0) if they are to be used for reliable TDT parameterization. The bottom line of this analysis is that, firstly, for the modeling it is fine to choose T_{c*} at a benign (rearing) temperature, as the choice has negligible impact on the model output as long as T_{c*} < true T_c. Secondly, if ramp experiments are to be used for TDT parameterization, we recommend the use of relatively high (to save time), but non-damaging temperatures, i.e. choose for example a start temperature in the higher spectrum of viable rearing temperatures for the species.

Guide to R-scripts

As a practical application of the mathematical framework presented in the main text, we here provide R-scripts to derive parameters of the thermal death time (TDT) curve and use these to assess thermal tolerance limits. We present two scripts (**Fig. S2**). Which one you should use depend on the type of input data which depends on the type of experiment conducted:

1) **"TDT_from_Static.R"**. This script derives TDT parameters from static experiments, where time to failure (t_{coma}) is measured at one or more constant temperatures. An input data template is provided (static_input.csv)

2) **"TDT_from_Dynamic.R"**. This script derives TDT parameters from dynamic experiments, where the maximal temperatures tolerated (dynamic CT_{max}, dCT_{max}) are measured using one or more ramping rates. An input data template is provided (dynamic_input.csv).

Both scripts use the derived TDT parameters to convert between and within static and dynamic measurements and input data of natural temperature fluctuations can be added to assess when failure occurs based on the derived TDT parameters.

In the contents below, you can click on the appropriate section depending on your type of data for details on the derivation of TDT parameters. Once the TDT parameters have been derived from either static or dynamic input data, you have four options within each script depending on which kind of output is wanted (**Fig. S2**).

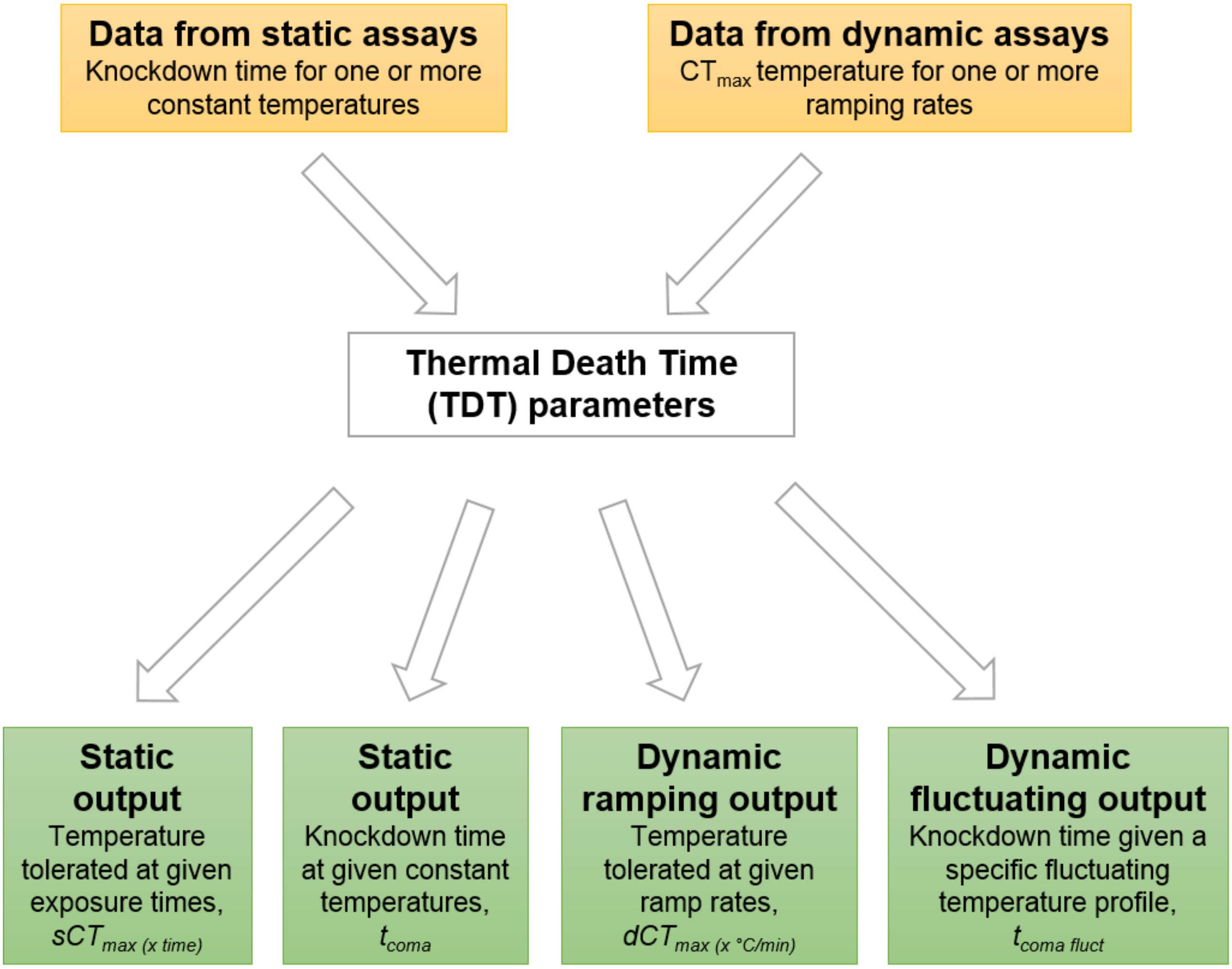

Fig. S2. Schematic workflow of scripts that derive TDT parameters and then allow conversion between and within static and dynamic output data along with prediction of injury accumulation under fluctuating temperatures.

Guide contents

1 TDT from static input data 6
1.1 Output: Tolerable temperature at a given exposure time 7
1.2 Output: Knockdown time at a given temperature 8
1.3 Output: Dynamic CT_{max} in ramping assays 9
1.4 Output: Knockdown time under randomly fluctuating temperatures 11
2 TDT from dynamic input data 12
2.1 Output: Tolerable temperature at a given exposure time 15
2.2 Output: Knockdown time at a given temperature 15
2.3 Output: Dynamic CT_{max} in ramping assays 16
2.4 Output: Knockdown time under randomly fluctuating temperatures 17

1 TDT curve from static input data

The script **"TDT_from_Static.R"** derives TDT parameters from static experiments, where time to failure (t_{coma}) is measured at one or more static temperatures. The corresponding input data template **"static_assay.csv"** contains three columns:

group: Identity label, e.g. treatment group or species binomial name if multiple species have been assessed.

t_coma: Time to failure [in minutes], e.g. time to onset of muscle loss. This can be the mean or median time of individuals assessed in each group at a given temperature.

assay_temp: Temperature [in °C] of the static experiment.

For each unique input group ID, the script will fit the TDT curve as a linear regression:

$$\log10(t_{coma}) = \beta \cdot \text{assay\_temp} + \alpha$$

Where β and α is the slope and intercept, respectively. From the slope, the thermal sensitivity coefficient, *z* is calculated as $z = -1/\beta$. The intercept with the y-axis (i.e. when temperature = 0 °C) has no biological relevance for heat stress, and instead we see this as "a point on the line" which can be substituted by another value. In the main manuscript we present $sCT_{max(1h)}$ (temperature that causes heat failure after a 1-hour exposure) as such an alternative value that, together with *z*, convey the same information of the TDT curve. The TDT curve is plotted for each unique group to visualize the spread around a linear relationship and R^2 is provided (see **Fig. S3** for an example of the graphic output).

In the following, the numbered headings of each output type correspond to the same numbers given at the start of the script when selecting desired output types

BOX 1

In the case t_{coma} has only been assessed at one constant temperature, you must provide a guess of the value of *z*. For invertebrates and fishes, values of *z* in the range 1 to 5 is a reasonable starting point (see **Table S1**). Either supply a general value for all groups or make it group specific with a vector, where *z* can be called for each group.

CAUTION: The estimate of *z* has extreme consequences for model predictions and excessive extrapolations from the original data point should be treated with considerable caution (see discussion in the main text)

1.1 Output: Tolerable temperature at a given exposure time

The script can use TDT parameters to predict sCT_{max} [in °C] for other exposure durations (t_{coma}), e.g. if you want to know what temperature your organisms can survive for e.g. 1 h ($sCT_{max(1h)}$) or 1 week ($sCT_{max(1week)}$). The desired additional exposure durations can be provided in the object 'extra_t_coma' [in minutes], note that 1 day = 1440 minutes. sCT_{max} for each supplied extra t_{coma} is determined from the TDT parameters β and α:

$$\mathrm{sCT}_{\mathrm{max}\,(\mathrm{t}_{\mathrm{coma}})} = \frac{\log 10(\mathrm{t}_{\mathrm{coma}}) - \alpha}{\beta}$$

Alternatively, if a point on the line (e.g. $sCT_{max(1h)}$ with t_{coma} = 1 h) and *z* is available, a similar calculation can be made:

$$\mathrm{sCT}_{\mathrm{max}\,(\mathrm{t}_{\mathrm{coma}})} = \mathrm{sCT}_{\mathrm{max}\,(1\mathrm{h})} - \mathrm{z} \cdot \log_{10}\left(\frac{\mathrm{t}_{\mathrm{coma}}}{\mathrm{t}_{\mathrm{coma}(1\mathrm{h})}}\right)$$

Where $sCT_{max(1h)}$ and the corresponding $t_{coma(1h)}$ can be substituted by any point on the line, including the intercept (0,α).

If additional t_{coma} times are provided, a csv table named "extra_t_coma.csv" is produced containing the input data along with the predicted sCT_{max} at the given t_{coma} times.

CAUTION: Severe extrapolation outside the time and temperature domain that was used to parameterize the TDT curve increases uncertainty of predictions, i.e. if observed t_{coma} is within minutes to hours, the model cannot confidently predict sCT_{max} in tests that last days (See main text).

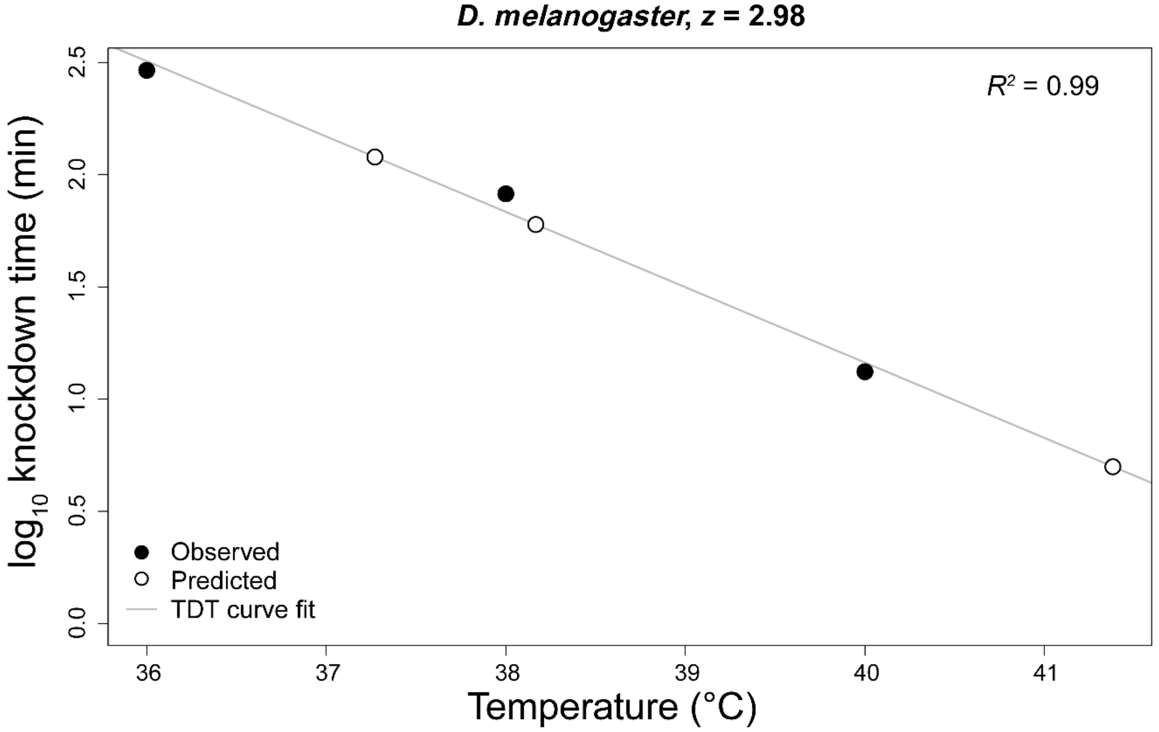

Fig. S3. Example of the graphic output of the "TDT_from_Static.R" script, which plots observed static tolerances (filled circles). Here we show an empirical example with *Drosophila melanogaster* from Jørgensen et al. (2019b), with three observed t_{coma} durations at 36, 38, and 40 °C (t_{coma} = 292.0, 82.25, and 13.25 minutes, respectively). The TDT curve is fitted as the regression of log10(t_{coma}) on assay temperature (grey solid line), and the R^2 value is provided. From the slope of the regression, *z* is calculated (z = -1/slope) and is provided in the plot title to allow an easy comparison of species-specific *z* values. If additional t_{coma} times are provided (section 1.1) or additional temperatures are provided (section 1.2), these will be predicted from the TDT parameters and plotted alongside the observed static tolerances (open circles, note they are positioned exactly on the TDT curve). Here we provide an example of three additional t_{coma} durations (5, 60, and 120 minutes), for which the tolerable temperature is predicted (41.38, 38.17, and 37.27 °C, respectively)

1.2 Output: Knockdown time at a given temperature

The script can use TDT parameters to predict t_{coma} [in minutes] at other static temperatures, e.g. if you want to know how long your organisms can survive at a stressful static temperature. The desired extra static temperatures can be provided in the object 'extra_sCTmax' [in °C]. The model can only handle positive temperatures (but in cases where cold stress is considered and subzero temperatures are relevant it is easy to convert all measures to the kelvin scale, however models assumptions and accuracy have not presently been tested for cold stress). t_{coma} for each additionally supplied sCT_{max} is determined from the TDT parameters β and α:

$$t_{coma} = 10^{\alpha+(\beta \cdot sCT_{max})}$$

Alternatively, if a point on the line (e.g. $sCT_{max(1h)}$ with t_{coma} = 1 h) and *z* is available, a similar calculation can be made:

$$t_{coma} = t_{coma(1h)} \cdot 10^{\frac{sCT_{max\,(1h)} - sCT_{max(t_{coma})}}{z}}$$

Where $sCT_{max(1h)}$ and the corresponding $t_{coma(1h)}$ can be substituted by any point on the line, including the intercept (0,α).

If additional temperatures are provided, these will also be plotted in the TDT curve plot alongside observed static tolerances (see **Fig. S3** for an example), and a csv table named "extra_sCTmax.csv" is produced containing the input data along with the predicted t_{coma} at the given sCT_{max} temperatures.

CAUTION: Severe extrapolation outside the time and temperature domain that was used to parameterize the TDT curve increases uncertainty of predictions (see main text).

1.3 Output: Dynamic CT_{max} in ramping assays

The script can use TDT parameters to predict dCT_{max} in dynamic assays where temperature is changed at a constant rate (ramp rate). In the object 'ramprates', you can supply the rates of temperature change [in °C/min] for which dCT_{max} should be predicted. Rates often range between 0.01 to 1.00 °C/min, but any positive numerical input is allowed.

CAUTION: consider the use of very slow or very fast ramping rates in the model only with caution as time or thermal equilibrium may be problematic (See main text and section 1.1).

The parameters used to estimate dCT_{max} are:

z: temperature sensitivity coefficient [dimensionless] from TDT curve or provided (see Box 1)

t_{Ls}: the time where the critical amount of injury has accumulated resulting in coma, i.e. t_{coma}, time of static CT_{max} (sCT_{max}) [min]

sCT_{max}: sCT_{max} [°C] at t_{Ls} (t_{coma})

T_0: ramp start temperature [°C]

T_{c^*}: T_c is the temperature above which net injury accumulation starts. As the 'true' T_c is rarely known, we recommend using some convenient value T_{c^*} below the true T_c, e.g. the rearing temperature. See main text for a discussion on this and **Fig. S1** for further justification.

The script sets t_{Ls} to the highest t_coma and the corresponding sCT_{max} is used. Then for each ramping rate, *b*, dCT_{max} [in °C] is estimated (equation 7a in main text):

$$dCT_{max} = T_0 + \frac{z}{\ln(10)} \ln\left[\frac{\ln(10) \cdot b \cdot t_{Ls}}{z} \cdot e^{\frac{\ln(10)}{z}(sCT_{max} - T_0)} + e^{\frac{\ln(10)}{z}(T_{c*} - T_{0})}\right]$$

Here we substituted *k* in equation 7a with ln(10)/*z*. Note that in *R* log() is the natural logarithm, whereas log10() is the base 10 or “common” logarithm.

If other ramp rates are provided, a csv table named “dCTmax_predictions.csv” is produced with the predicted dCT_{max} for each supplied ramp rate.

1.4 Output: Knockdown time under randomly fluctuating temperatures

From the TDT parameters (β and α) estimated from the static assays (observed sCT_{max} temperatures and t_{coma} durations), the script can predict time to failure for experiments in which temperature fluctuates either randomly or predictably, from a specific fluctuating temperature profile. An input template is provided ("fluctuating_temperature_profile.csv"). Time must be provided in minutes, e.g. 10 seconds as 1/6 min, and temperature at that time must be supplied in °C.

Assuming additivity of thermal injury, time to failure is predicted based on the sum of accumulated thermal injury:

$$\text{Accumulated injury} = \sum_{i=1}^{t_e} \frac{100 \cdot (t_{i+1} - t_i)}{10^{(\beta \cdot \max(T_i;T_{i+1})+\alpha)}}$$

Which sums the additive injury over each time interval *i* until t_e which is the time interval for which the total accumulated injury is calculated. The accumulated injury for which the tolerable exposure duration should be predicted (corresponding to t_e, the time interval where summation of cumulative injury should stop) can be set to any percent lethal damage above 0 and up to 100. In the case of 100 % accumulated injury t_e equals t_{coma}. At each time step t_i, the interval in minutes until the next measurement is determined regardless of whether temperatures have been recorded with equal intervals. The denominator is the fraction of the tolerable exposure duration for the maximum temperature (of T_i and T_{i+1}) in each time interval. The time where accumulated injury is closest to the set percent lethal damage is returned for each group provided. If a fluctuating temperature profile is provided a csv table named "fluctemp_predictions.csv" is produced.

CAUTION: Temperatures below the damage accumulation threshold might "repair" thermal injury (see main text).

2 TDT curve from dynamic input data

The script **"TDT_from_Dynamic.R"** derives TDT parameters from dynamic experiments, where temperature is changed at a constant rate (ramp rate) and the maximal temperatures tolerated (dCT_{max}) are measured using one or more ramping rates. The corresponding input data template **"dynamic_input.csv"** contains three columns:

group: Identity label, e.g. treatment group or species binomial name if multiple species have been assessed

ramprate: Temperature change [in °C/min]

dCT_{max}: Temperature at time of failure [in °C] of the dynamic experiment

The parameters used to estimate sCT_{max} are:

t_{Ls}: The time where the critical amount of injury has accumulated resulting in coma, i.e. t_{coma}, time of static CT_{max} (sCT_{max}) [min] given in the object 't_coma' (see below)

ramp rate: Temperature change [in °C/min]

dCT_{max}: Temperature at time of failure [in °C] at a given ramp rate

T_0: ramp start temperature [°C]

T_{c*}: T_c is the temperature where damage accumulation starts. As the 'true' T_c is rarely known, however, we recommend using some convenient value T_{c*} below the true T_c (e.g. the rearing temperature). See main text for a discussion on this and **Fig. S1** for further justification.

NOTE: In order to get a starting point from which to build the TDT curve, at least one t_{coma} value MUST be supplied (a point on the line) in the object 't_coma'. The script will stop and print an error message if it is not provided. We recommend a t_{coma} value of 1 h, at this will accommodate the duration of most thermal assays. If you have dynamic assays than span a duration much shorter or much longer than 1 h, consider using the average duration as a starting t_{coma} point for parameterizing the TDT curve. If several tolerable static temperatures should be predicted as the output (section 2.1), you can supply several t_{coma} values in this object [in mins]. The script will derive TDT parameters and predict sCT_{max} for each of the supplied t_{coma} (t_{Ls}) durations. There are three different methods depending on how many ramp rates dCT_{max} has been assessed for:

From dCT_{max} at three or more ramp rates

In the case of three or more available ramp rates, the script fits a non-linear model on dCT_{max} and the corresponding ramp rate *b* via the nonlinear least squares (nls) function in R to estimate *z* and sCT_{max} for each supplied t_{coma} duration (equation 7a in main text):

$$dCT_{max} = T_0 + \frac{z}{\ln(10)} \ln\left[\frac{\ln(10) \cdot b \cdot t_{Ls}}{z} \cdot e^{\frac{\ln(10)}{z}(sCT_{max} - T_0)} + e^{\frac{\ln(10)}{z}(T_{c*} - T_0)}\right]$$

Here we substituted *k* in equation 7a with ln(10)/*z*. Note that in *R* log() is the natural logarithm, whereas log10() is the base 10 or "common" logarithm. Also note, *z* is independent of t_{coma} (t_{Ls}). nls() iterates from a user supplied starting position for each variable, with the argument 'start = list(sCTmax=35, z=2.5)' (example starting values). Note the order of variables in this list must be maintained. This can be made group specific with a starting value vector before the loop over groups and calling the individual values at each iteration, i, but generally nls performs well with reasonable guesses across groups.

From dCTmax at two ramp rates

In the case of two ramp rates, the script sets up two equations with two unknowns with the two ramp rates and corresponding values of dCT_{max} and solves for *z* and sCT_{max} with the 'rootSolve' R package (this is installed and loaded initially if not already present; Soetaert 2009; Soetaert & Herman 2009). The multiroot() function must be supplied with starting values (start guesses) with the argument 'start = c(2.5,35)' (example starting values for *z* and sCT_{max}, respectively, note the order of variables must be maintained). Only positive solutions are allowed.

From dCTmax at one ramp rate

In the case where dCT_{max} has been determined in an experiment with only a single ramp rate, you need to supply a value of *z*. CAUTION: The estimate of z has extreme consequences for model predictions and excessive extrapolation from the original data-point should treated with considerable consideration (see discussion in the main text and Box 1 for advice and considerations on selecting appropriate values of *z*)

sCT_{max} is determined for each t_{coma} duration (tLs) (equation 7b in main text):

$$sCT_{max} = T_0 + \frac{z}{\ln(10)} \mathrm{Ln}\left[\frac{z}{\ln(10) \cdot b \cdot t_{Ls}}\left(e^{\frac{\ln(10)}{z}(dCT_{max} - T_0)} - e^{\frac{\ln(10)}{z}(T_{c*} - T_0)}\right)\right]$$

Here we substituted *k* with ln(10)/*z*. Note that in *R* log() is the natural logarithm, whereas log10() is the base 10 or "common" logarithm.

For each unique group, dCT_{max} is plotted as a function of ramp rate to visualize the goodness of fit. If additional dynamic tolerances are desired (see section 2.3 below), these will also be plotted in the same plot (see **Fig. S4** for an example of the graphic output).

In the following, the numbered headings of each output type correspond to the same numbers given at the start of the script when selecting desired outputs. Note that Output 2.1: Tolerable temperature at a given exposure time, is not optional as t_{coma} must be supplied (see above).

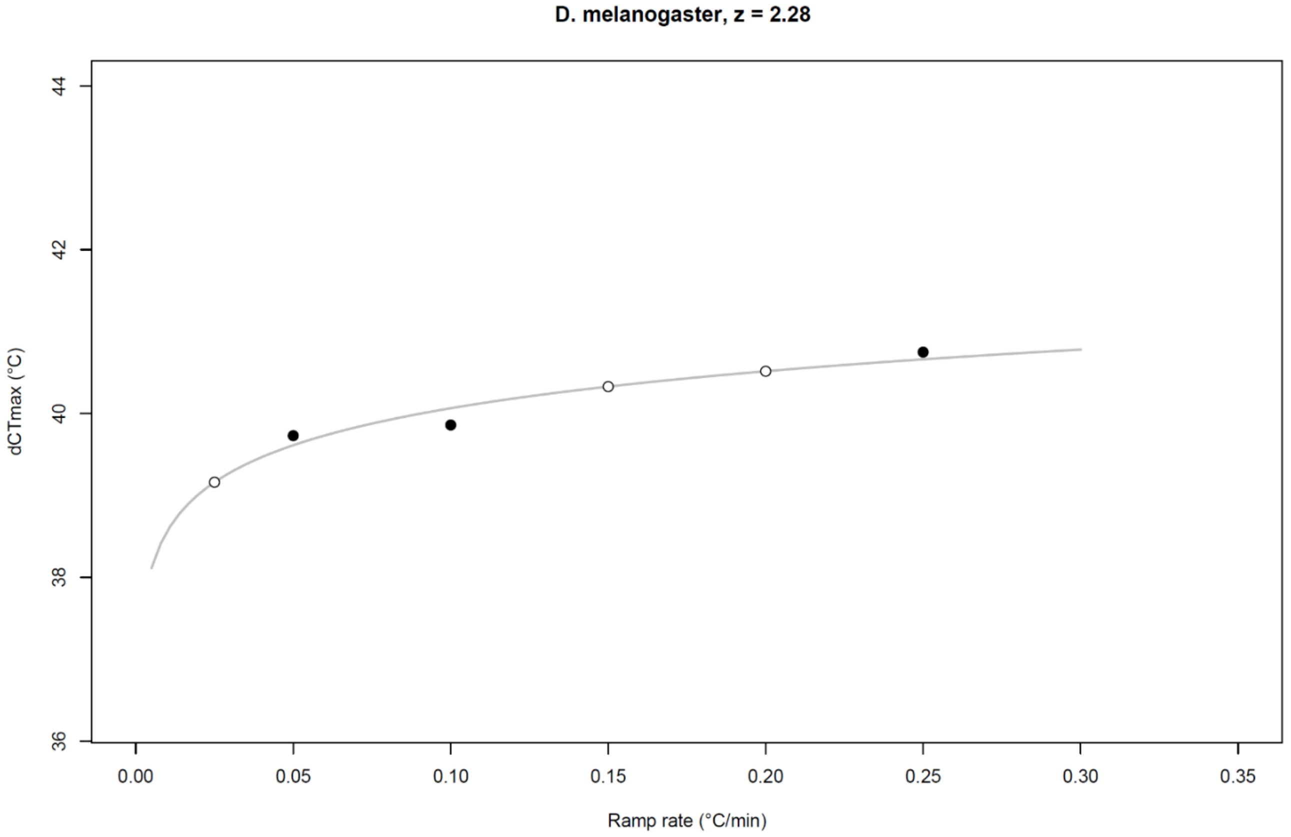

Fig. S4. Example of the graphic output of the "TDT_from_Dynamic.R" script, which plots observed dynamic tolerances (filled circles), as a function of ramp rate [°C/min]. Here we show an empirical example with *Drosophila melanogaster* from Jørgensen et al. (2019b), with three observed dynamic CT_{max} (dCT_{max}) temperatures from experiments employing ramp rates of 0.05, 0.10, and 0.25 °C/min (dCT_{max} = 39.73, 39.86, and 40.75°C, respectively). The grey solid line represents the fitted relationship between dCT_{max} and ramp rate (in this example via nonlinear least squares (nls) function). The derived value of *z* is provided in the plot title to allow an easy comparison of species-specific values of *z*. If additional ramp rates are provided (section 2.3), dCT_{max} at these rates will be predicted from the TDT parameters and plotted as well (open circles, note they are positioned exactly on the dynamic TDT curve). Here we provide an example of three additional ramp rates (0.025, 0.15, and 0.20 °C/min), for which dCT_{max} is predicted (39.16, 30.33, and 40.52°C, respectively).

2.1 Output: Tolerable temperature at a given exposure time

The script can use TDT parameters to predict sCT_{max} in static assays, with constant temperature exposure. In the object 't_coma', you can supply a range of exposure durations [in minutes] for which sCT_{max} should be predicted (note that for TDT curve parameterization from dynamic assays already at least one t_{coma} has been supplied, see section 2 TDT curve from dynamic input data). Any positive input is allowed.

CAUTION: Severe extrapolation outside the time and temperature domain that was used to parameterize the TDT curve increases uncertainty of predictions, i.e. if observed t_{coma} is within minutes to hours, the model cannot confidently predict sCT_{max} in tests that last days.

Regardless of the method for estimating sCT_{max} and *z* (see the three methods above), the output of the script is a csv table named "sCTmax_predictions.csv" with the predicted sCT_{max} and *z* for each t_{coma} for each unique group ID.

2.2 Output: Knockdown time at a given temperature

The script can also use TDT parameters to predict t_{coma} times [in minutes] at other static temperatures, e.g. if you want to know how long your organisms can survive at a specific stressful temperature. The desired additional static temperatures can be provided in the object 'extra_sCTmax' [in °C]. The model can only handle positive temperatures (but in cases where cold stress is considered and subzero temperatures are relevant it is easy to convert all measures to the kelvin scale, however models assumptions and accuracy have not presently been tested for cold stress). t_{coma} for each additionally supplied sCT_{max} is determined from the TDT parameters β and α:

$$t_{coma} = 10^{\alpha+(\beta \cdot sCT_{max})}$$

Alternatively, if a point on the line (e.g. $sCT_{max(1h)}$ with t_{coma} = 1 h) and *z* is available, a similar calculation can be made:

$$t_{coma} = t_{coma(1h)} \cdot 10^{\frac{sCT_{max\,(1h)} - sCT_{max(t_{coma})}}{z}}$$

Where $sCT_{max(1h)}$ and the corresponding $t_{coma(1h)}$ can be substituted by any point on the line, including the intercept (0,α).

CAUTION: Severe extrapolation outside the time and temperature domain that was used to parameterize the TDT curve increases uncertainty of predictions, i.e. if observed sCT_{max} is within 36-40 °C, the model cannot confidently predict t_{coma} for temperatures far outside this range, e.g. 50 °C.

If additional temperatures are provided, a csv table named “extra_sCTmax.csv” is produced containing the predicted sCT_{max} for each supplied t_{coma} (see section 2.1) along with the predicted t_{coma} times at additionally supplied sCT_{max} temperatures.

2.3 Output: Dynamic CT_{max} in ramping assays

The script can predict dCT_{max} for additional ramp rates not included in the input data. In the object ‘extra_ramprates’, you can supply additional rates of temperature change [in °C/min] for which dCT_{max} should be predicted. Rates often range between 0.01 to 1.00 °C/min, but any positive numerical input is allowed.

CAUTION: consider the use of very slow or very fast ramping rates in the model only with caution as time or thermal equilibrium may be problematic (See main text and section 1.1).

From the derived *z* and sCT_{max} at given t_{coma} times (at least one), dCT_{max} is predicted for each supplied ramp rate (equation 7a in main text):

$$dCT_{max} = T_0 + \frac{z}{\ln(10)} \ln\left[\frac{\ln(10) \cdot b \cdot t_{Ls}}{z} \cdot e^{\frac{\ln(10)}{z}(sCT_{max} - T_0)} + e^{\frac{\ln(10)}{z}(T_{c^*} - T_{0})}\right]$$

where *b* is the ramp rate. Here we substituted *k* in equation 7a with ln(10)/*z*. Note that in *R* log() is the natural logarithm, whereas log10() is the base 10 or “common” logarithm.

If additional ramp rates were provided, a csv table named “dCTmax_extra_ramprates.csv” is produced containing the predicted dCT_{max} for each supplied ramp rate.

2.4 Output: Knockdown time under randomly fluctuating temperatures

From the TDT parameters (β and α) estimated from the dynamic ramping assay (predicted sCT_{max} at specified t_{coma} durations), time to failure can be predicted for experiments in which temperature fluctuates either randomly or predictably. If desired, you must provide a fluctuating temperature profile. An input template is provided ("fluctuating_temperature_profile.csv"). Time must be provided in minutes, e.g. 10 seconds must be given as 1/6 min, and temperature at that time must be supplied in °C.

Assuming additivity of thermal injury, time to failure can be predicted based on the sum of accumulated thermal injury:

$$\text{Accumulated injury} = \sum_{i=1}^{t_e} \frac{100 \cdot (t_{i+1} - t_i)}{10^{(\beta \cdot \max(T_i; T_{i+1}) + \alpha)}}$$

Which sums the additive injury over each time interval *i* until t_e which is the time interval for which the total accumulated injury is calculated. The accumulated injury for which the tolerable exposure duration should be predicted (corresponding to t_e, the time interval where summation of cumulative injury should stop) can be set to any percent lethal damage above 0 and up to 100. In the case of 100 % accumulated injury t_e equals t_{coma}. At each time step t_i, the interval in minutes until the next measurement is determined regardless of whether temperatures have been recorded with equal intervals. The denominator is the fraction of the tolerable exposure duration for the maximum temperature (of T_i and T_{i+1}) in each time interval. The time where accumulated injury is closest to the set percent lethal damage is returned for each group provided. If a fluctuating temperature profile is provided a csv table named "fluctemp_predictions.csv" is produced.

CAUTION: Temperatures below the damage accumulation threshold might "repair" thermal injury. (see main text).

Table S1. Overview of the values of *z* used for each species in cases where only a single data entry was available for TDT parameterization. These mean values of *z* for each species are based on TDT parameterization where sufficient (i.e. at least two) data entries were available and may thus include both TDT curves based on static and dynamic data. The range of the values of parameterized *z* is given along with the number (n) of TDT curves that the mean value of *z* is calculated from (this number corresponds to the number of triangle points in Fig. 5 in the main text).

**Group**	**Species**	**Mean parameterized z**	**z range**	**n**
Insects	*Drosophila melanogaster*	2.55	1.5 - 3.1	7
	Drosophila subobscura	3.40	2.6 - 4.7	9
	Glossina pallidipes	5.15	4.7 - 5.8	3
	Tenebrio molitor	0.90	-	1
Springtails	*Orchesella cincta*	1.80	1.8 - 1.8	2
	Folsomia candida	3.30	-	1
Waterfleas	*Daphnia magna*	2.20	0.8 - 3.4	3
Fishes	*Gambusia affinis*	1.80	1.1 - 2.4	7
	Salmo salar	1.78	1.4 - 2.2	8
	All species	2.54	0.8 - 5.8	41

Table S2. Summary table for *F*-test between linear regression of additivity fractions (Fraction at T_2 ~ Fraction at T_1) and the line of additivity (y-intercept = 1, slope = -1) (Fig. 3). *Category*, High temperature first (HF) or "low" temperature first (LF); *Model*, the tested model; *Residual Df*, degrees of freedom for each model; *RSS*, residual sum of squares; *Df*, degrees of freedom between the two tested models; *SS*, sum of squares; *F*, F-statistic; *P-value*, *P*-value.

**Category**	**Model**	**Residual *Df***	**RSS**	***Df***	***SS***	***F***	***P*-value**
HF_female	Line of additivity	16	0.037				
	Linear regression	14	0.027	2	0.010	2.689	0.103
HF_male	Line of additivity	16	0.078				
	Linear regression	14	0.061	2	0.017	1.999	0.172
LF_female	Line of additivity	18	0.261				
	Linear regression	16	0.259	2	0.002	0.092	0.912
LF_male	Line of additivity	18	0.547				
	Linear regression	16	0.428	2	0.119	2.215	0.142

Table S3. Summary table for *F*-test between linear regression of observed t_{coma} against predicted t_{coma} from experiments with fluctuating temperatures and the line of unity (y-intercept = 0, slope = 1) (Fig. 4). *Sex*, sex; *Model*, the tested model; *Residual Df*, degrees of freedom for each model; *RSS*, residual sum of squares; *Df*, degrees of freedom between the two tested models; *SS*, sum of squares; *F*, F-statistic; *P-value*, *P*-value.

**Sex**	**Model**	**Residual *Df***	**RSS**	***Df***	***SS***	***F***	***P*-value**
Female	Line of unity	13	3836				
	Linear regression	11	1859	2	1977	5.85	0.018
Male	Line of unity	13	3694				
	Linear regression	11	1079	2	2615	13.33	0.001

Supplementary references

Jørgensen, L.B., Malte, H. & Overgaard, J. (2019b). Data from: How to assess Drosophila heat tolerance: unifying static and dynamic tolerance assays to predict heat distribution limits. Dryad Digital Repository. https://doi.org/10.5061/dryad.840j728.

Soetaert, K. (2009). rootSolve: Nonlinear root finding, equilibrium and steady-state analysis of ordinary differential equations. R-package version 1.6.

Soetaert, K. & Herman, P. (2009). *A Practical Guide to Ecological Modelling. Using R as a Simulation Platform*. Springer, New York, NY, USA.
